# Institutional differences and geographical disparity: the impact of medical insurance on the equity of health services utilization by the floating elderly population - evidence from China

**DOI:** 10.1186/s12939-019-0998-y

**Published:** 2019-06-14

**Authors:** Junqiang Han, Yingying Meng

**Affiliations:** 1 School of Public Management, South-Central University for Nationalities, Wuhan, 430074 China; 2 Center for Social Security Studies, Wuhan University, Wuhan, 430072 China

**Keywords:** Medical insurance, Health service, Inequity, The floating population, China, The basic medical insurance system for urban employees, The basic medical insurance system for urban and rural residents

## Abstract

**Background:**

The Chinese government has now achieved universal coverage of medical insurance through two systems: the Basic Medical Insurance System for Urban Employees (BMISUE) and the Basic Medical Insurance System for Urban and Rural Residents (BMISURR). This paper aims to identify the impact of China’s current medical insurance system on equity in the use of health services by the floating elderly population from two aspects: institutional differences and geographical disparity.

**Methods:**

The data used in the study are from the China Migrants Dynamic Survey (CMDS) conducted by the National Health and Wellness Council of China. This study uses the Logit model to estimate the impact of the medical insurance system on the utilization of health services and the Propensity Score Matching (PSM) method to further test the robustness of the results.

**Results:**

The study found that the type of medical insurance does not affect health services utilization by the floating elderly population in China. However, for those participating in the same medical insurance, participation in different regions will significantly affect the use of health service resources. For the BMISURR, when the place of the insurance is the same as the place of residence, the proportion of the floating elderly population that will see a doctor when they are sick will increase by 4.80%. For the BMISUE, when the place of the insurance is the same as the place of residence, the proportion of the floating elderly population that will see a doctor when they are sick will increase by 10.30%.

**Conclusions:**

The difference between the place of insurance and the place of residence results in the unbalanced utilization of health services by the floating elderly population participating in the same medical insurance system.

## Introduction

The medical insurance system is an integral part of the national public health system [1]. It helps reduce the burden of medical services for the insured by lowering the economic threshold for receiving medical services, thereby improving the health of the insured to a certain extent [2]. In recent years, the Chinese government has established a series of medical insurance systems to improve the health of its citizens, intending to build a national medical insurance system framework, improve the utilization rate of domestic health services and improve the overall health of the nation [3].

Since the implementation of the reform and opening up policy in the late 1970s, the Chinese government has built a system of universal medical insurance in a step-by-step manner [4]. In 1998, the combination of social pooling accounts and individual accounts was adopted for the employees of urban enterprises. The establishment of the Basic Medical Insurance System for Urban Employees (BMISUE) marks the transformation of China from the “welfare” medical insurance under the planned economic system to the “social insurance” medical insurance in which employers and employees share responsibility under the market economic system.

In 2003, the principle of voluntary participation was adopted for rural residents, and the new rural cooperative medical system (NRCMS) was established nationwide, which benefited rural residents, who accounted for more than 50% of the population, with basic health protection. In 2007, the principle of voluntary participation was adopted for the urban residents, and the basic medical insurance system for urban residents (BMISUR) was established nationwide. The protection offered by this system is mainly for flexible employees who do not participate in the BMISUE among urban residents, those who have reached working age, those who are not employed, students in primary and secondary schools, children, college students, and those with urban household registration, especially for rural migrants who work in cities.

Since the two systems of the BMISUR and NRCMS have gaps regarding reimbursement scope and payment level, the Chinese government has integrated these two systems since 2015 to realize the equity of the basic medical insurance system for urban and rural residents [5]. The basic medical insurance system for urban and rural residents (BMISURR) was thus established. The integrated urban and rural residents’ medical insurance system covers all urban and rural residents except for urban employees with basic medical insurance. In general, the Chinese government has now achieved universal coverage of medical insurance through the two systems of BMISUE and BMISURR.

Of the two main medical insurance systems in China, on the one hand, the BMISUE is a high-paid, high-guaranteed medical insurance system, while the BMISURR has a relatively low level of contribution, which means the level of protection is also relatively low. The institutional differences between these two medical insurance systems are the subject of this paper. How do the differences in the medical insurance systems affect the equity of the health service utilization of the participants?

China’s current medical insurance system is based on the prefecture-level city and county-level city as the basic pooling unit. That is, an insured person participates in the medical insurance system in units of prefecture-level and county-level cities and receives health services and compensation for insurance expenses. In the regulations of the current system, although insured persons are allowed to receive health services outside the participating regions, the local government has stipulated that the proportion of reimbursement for health service expenses generated in different pooling units is lower than that in the local unit, it is necessary to set up hospitals in different pooling units (other than the unit where the participating medical insurance management agency signs the cross-regional referral cooperation agreement); and various cumbersome certification materials must be provided for reimbursement. The current medical insurance system cannot be “carried” freely between different regions and there is a clear regional partition in the current medical insurance system. This paper defines this division as geographical disparity. Thus, does this geographical disparity also have an impact on the equity of the health service utilization by the insured?

This paper argues that the Chinese government has designed different types of medical insurance for different groups. If the difference in medical insurance type leads to differences in the utilization of residents’ health services, we think this represents inequity. At the same time, if residents participate in the same type of medical insurance but have different use of health services due to the different areas of participation, we think that this is a also kind of inequity. This paper will discuss the impact of the medical insurance system on the equity of the health service utilization of the participants from two aspects: institutional differences and geographical disparity.

## Literature review

Many scholars have investigated the impact of the medical insurance system on the utilization rate of health services. Many classic studies have discussed the positive effects of the medical insurance system on the utilization of health services. Newhouse, Manning [6] used RAND insurance experiments to find that consumers who were randomly assigned to a free medical plan had 46% more medical expenses than consumers with a 95% payout plan. Young and Cohen [7] studied patients admitted to the Massachusetts Hospital for acute myocardial infarction and found that after controlling the characteristics of individuals and hospitals, uninsured patients had significantly higher mortality after 30 days of discharge than patients with commercial medical insurance. Gaylin, Held [8] studied more than 4000 dialysis patients with end-stage renal disease in the USA, and it was found that uninsured patients had a late stage of diagnosis and a short survival time after discharge. Cheng and Chiang [9] studied the changes in demand for medical services after the introduction of universal medical insurance in Taiwan in China in 1995. Since universal medical insurance reduced the out-of-pocket amount and proportion of outpatient and inpatient medical care, the probability of medical treatment for uninsured people before 1995 doubled after the implementation of universal health insurance. Finkelstein, Taubman [10] studied the impact of the Oregon Health Plan (OHP) on the personal health of those 19–64 years old (OHP provides health care coverage for low-income Oregonians), and found that the plan can significantly improve the health service utilization and physical and mental aspects of the insured. According to the overall evaluation, most scholars agree that the medical insurance system can effectively help the insured face unpredictable economic conditions by reducing the economic threshold of medical treatment. By improving the utilization rate of health services of insured persons against the risk of medical expenditures, health insurance has a positive effect on the health of citizens.

Some studies have also paid attention to the impact of various medical insurance systems in China on the utilization rate of health services among participants [11, 12]. Some scholars have studied the impact of the NRCMS on the utilization of rural residents’ health services by comparing the policy environment where the NRCMS was launched in different places at different times. Wang, Yip [13] used the method of experimental research to compare the areas where the NRCMS was carried out to randomly selected undeveloped areas and found that the NRCMS significantly improved the utilization rate of health services among rural residents and reduced the proportion of self-reported pain and anxiety among rural residents of all ages. Zhou and Li [14] used the panel data of 2000 and 2006 in the China Health Nutrition Survey (CHNS) database to analyze the impact of the implementation of the NRCMS on the utilization of health services among rural middle-aged and elderly people and found that the implementation of the NRCMS improved the utilization rate of health services in rural areas, and generally improved the health status.

Some studies have focused on the impact of the BMISUR on the utilization of health services among urban residents. Hu and Liu [15] used the comprehensive utilization tendency score matching principle and double difference method to evaluate the impact of the BMISUR on the utilization rate of health services among urban residents and believed that this system mainly improves the lives of people with low-income and poor health. The research of Pan, Lei [16] shows that participating in the BMISUR significantly improves people’s health service utilization but does not increase the individual’s financial burden, is conducive to promoting the health of the insured, and affects the people with low socioeconomic status.

Other studies have focused on the impact of BMISUE on the utilization of urban workers’ health services. Chen and Deng [17] used the CHNS data for 2009 and 2011 to empirically analyze the impact of the BMISUE on the health status of participants in terms of short-term and long-term health status. The results show that the participation in the BMISUE has improved the short-term health status of the participants to a certain extent and can significantly improve the long-term health. At the same time, participating in the BMISUE will increase the actual medical expenses of the insured.

In summary, in the existing research on the impact of China’s medical insurance system on the equity of citizen health service utilization, scholars mainly focus on participation in a particular medical insurance system based on single dimensions to analyze the impact on the use of health services by participants of the BMISUE, BMISUR, BMISURR or NRCMS [14, 17–19]. There is no study comparing the differences in the effects of different current medical insurance systems in China on the equity of health service utilization among insured persons. Moreover, there is no research on the impact of the geographical segmentation of the system on the equity of the health service utilization of the insured.

In reality, the inequitable use of health services caused by institutional differences and geographical disparity is particularly prominent for the floating population. The sharp increase in population migration is one of the most prominent features of China’s population development in this century. According to data from the National Bureau of Statistics, China’s floating population reached 245 million in 2017. These floating populations change their workplaces frequently between villages and cities or between different cities to obtain jobs. The mobility often leads to inconsistency between the locations where they participate in the medical insurance system and where they receive health services. This study takes the elderly population, with relatively high health service demand, among the floating population as the research object, and uses the data of the CMDS in 2015 to analyze the impact of medical insurance on the equity of the utilization of health services from two aspects: institutional difference and geographical disparity.

## Methods

### Data source

The data used in this paper are from the 2015 CMDS, which is derived from the annual large-scale national sample of migrant population surveys conducted by the National Health and Health Council of China since 2009. In 2015, the CMDS surveyed male and female migrants aged 15 and older who had not lived in the local area (city, county) for 1 month or more, covering 31 provinces (autonomous regions, municipalities) in 2014. The current population data of the floating population is the primary sampling frame, and the stratified, multistage, and Probability Proportionate to Size (PPS) sampling method is used for sampling. The PPS method is the abbreviation of proportional probability sampling based on size. It is a sampling method that uses auxiliary information to give each unit the probability to be selected proportionally according to its size.

In the first stage, the township streets were selected according to the PPS method; in the second stage, the village residents’ committees were chosen according to the PPS method in the township streets where they were drawn; in the third stage, the individual investigation objects were selected in the neighborhood committees (village committees). In 2015, the CMDS aimed to investigate the status of the survival of the floating population and the utilization of public health services by the elderly (defined as 60 years old and above, when the questionnaire was completed, those born before 1955), of those surveyed 4484 were elderly. This article aims to analyze the impact of the medical insurance system on the equity of the use of health services for the floating elderly population.

Among them, the definition of the floating elderly population is living in the place of data collection for 1 month or more, having non-local (city, county) household registration, and being older than or equal to 60 years old (when filling out the questionnaire, the birth-year-old male and female before the month of May 1955). It should be noted that the household registration system is a unique population registration management system in China. When a citizen is born, he or she is given a household registration status according to the administrative jurisdiction (city, county or township) where he was born. If an individual moves to another jurisdiction, he or she can choose whether to change his or her household registration. Therefore, according to whether there is a difference between the place of household registration and the place of data collection, that is, the site of official residence (the place where the long-term residence is), this paper divides the places where the floating elderly population participates in the medical insurance system into the place of residence and non-residence.

### Variables

#### Measurement of independent variables: the status of the floating elderly population’s participation in medical insurance

For the floating elderly population, participating in different medical insurance systems and participating in the same medical insurance system in different locations may affect the utilization of health services. Therefore, in this paper, we not only distinguish between different medical insurance systems but also separate the places where the medical insurance system is involved.

Table 1 shows the medical insurance participation of the sampled floating elderly population. Table 1 shows that the proportion of those participating in the BMISURR (in non-residential places) is the highest, reaching 50.71%; the proportion of participating in the BMISUE (in residence places) is the lowest, only 2.19%; and 16.46% of the floating elderly population is unclear or does not participate in medical insurance.

**Table 1:** Sample distribution of China’s floating elderly population participating in medical insurance status

Social insurance status	BMISURR (in residence places)	BMISURR (in non-residential places)	BMISUE (in residence places)	BMISUE (in non-residential places)	Unclear or not participating in medical insurance	In total
No.	360	2274	98	1014	738	4484
Proportion	8.03%	50.71%	2.19%	22.61%	16.46%	100%

*BMISURR* Basic Medical Insurance System for Urban and Rural Residents, *BMISUE* Basic Medical Insurance System for Urban Employees

#### Measurement of the dependent variables: the use of health services by the floating elderly population

In previous studies, the utilization of health service was measured by the two-week prevalence rate and hospitalization rate. In this study, the question of whether an individual would go to the hospital when sick is used to measure the utilization of health services by the floating elderly population.

The revelvant question in the 2015 CMDS questionnaire is: “If you are sick, how do you usually deal with?” When the participants choose “see a doctor”, the assignment is 1. When the participants choose “Buy medicine at a local pharmacy”, “ Take medicine from the home”, “Do nothing, wait for self-healing” or other options, the value is 0. As shown in Table 2, when the participant is sick, 42.17% of the elderly surveyed choose to see a doctor, and 57.83% choose another treatment.

**Table 2:** Sample distribution of the status of health services utilization by the floating elderly population of China

Treatment method	See a doctor	Buy medicine at a local pharmacy	Take medicine from the home	Do nothing, wait for self-healing	Others	In total
No.	1891	2475	25	39	54	4484
Proportion	42.17%	55.20%	0.56%	0.87%	1.20%	100%

#### Controlled variables

In addition to the categories of medical insurance, in this study, we controlled for individual characteristics of the floating elderly population, such as gender, age, ethnicity, household registration (hukou), marital status, education level, monthly household income in the empirical research process. Other variables that may influence the utilization of individual health services are controlled for as well, such as range of migration, main source of income, number of friends at the place of residence, daily exercise time, and take physical examination.

The economic and social development of different provinces and cities may affect the utilization of health services of residents. Furthermore, the medical insurance policies currently implemented in different provinces and cities are different in aspects such as medical insurance reimbursement standards and where reimbursement is recorded. This may also affect the use of different medical insurance systems by residents. We tried to minimize the influence of these differences by controlling for place of residence (the place where the floating elderly population participates in the medical insurance system). The definitions of the controlled variables and descriptions of the percentage or mean are shown in Table 3.

**Table 3:** Definitions of controlled variables and description of the mean

Variable name		Variable definition	Percentage/ Mean
	Gender	Dummy variable: male = 1, female = 0	1 = 60.75%, 0 = 39.25%
	Age	Continuous variable: age of the participant	Mean = 66.2507
	Ethnic	Dummy variable: Han ethnicity = 1, ethnic minorities = 0	1 = 90.86%, 0 = 9.14%
Type of Household Registration (Hukou)	Rural	Dummy variable: rural hukou = 1, others = 0	1 = 60.10%
	Nonrural hukou	Dummy variable: nonrural hukou = 1, others = 0	1 = 38.05%
	Rural to resident hukou	Dummy variable: rural to resident hukou = 1, others = 0	1 = 1.23%
	Nonrural to resident hukou	Dummy variable: nonrural to resident hukou = 1, others = 0	1 = 0.62%
Marital Status	Single	Dummy variable: single = 1, others = 0	1 = 0.91%
	Married	Dummy variable: married = 1, others = 0	1 = 80.62%
	Divorce or widowed	Dummy variable: divorced/widowed = 1, others = 0	1 = 18.47%
	Education level	Categorical variable: unschooled = 1, primary school = 2, middle school = 3, high school or technical secondary school = 4, postsecondary college = 5, university = 6, postgraduate = 7	1 = 13.38%, 2 = 38.72%, 3 = 29.86%, 4 = 12.11%, 5 = 3.84%, 6 = 2.07%, 7 = 0.02%。
Range of Migration	Interprovince	Dummy variable: participant migrated from one province to another = 1, otherwise = 0	1 = 42.04%
	Intercity	Dummy variable: participant migrated from one city to another within the same province = 1, otherwise = 0	1 = 31.47%
	Intercounty	Dummy variable: participant migrated from one county to another within the same province = 1, otherwise = 0	1 = 26.49%
	Monthly household income	Continuous variable: monthly household income of the participant (after tax)	Mean = 4975.873
Main Source of Income	Employment	Dummy variable: participant’s main source of income is his/her own employment = 1, otherwise = 0	1 = 30.73%
	Pension/Savings	Dummy variable: participant’s main source of income is pension or savings = 1, otherwise = 0	1 = 42.22%
	Other family members	Dummy variable: participant’s main source of income is from other family members = 1, otherwise = 0	1 = 22.48%
	Others	Dummy variable: participant’s main source of income is from none of the three aforementioned categories = 1, otherwise = 0	1 = 4.57%
	No. of friends at place of residence	Continuous variable: no. of friends at place of residence	Mean = 8.4683
	Daily exercise time (min)	Continuous variable: average time spent exercising daily, in minutes	Mean = 66.0203
	Physical examination	Dummy variable: participant has had at least one physical examination in the past year = 1, otherwise = 0	1 = 35.62%, 0 = 64.38%
	Self-rated health	Categorical variable: very unhealthy = 1, unhealthy = 2, basic health values = 3, health values = 4	1 = 0.91%, 2 = 8.70%, 3 = 44.45%, 4 = 45.94%

### Measurement model

This article uses whether people would go to the hospital when they are sick (1 is would go to the hospital, 0 is would not go to the hospital) to measure the utilization of health services by the floating elderly population. The variable value is 0 or 1, so this article uses the logit model to estimate the impact of the insurance system on the utilization of health services. The measurement model is as follows:

$$ \log \frac{P_i}{1-{P}_i}={\beta}_0+{\beta}_1{Insurance}_i+{\beta}_2{Person}_i+{\beta}_3{Control}_i+{\varepsilon}_i $$

where *P*_*i*_ is the probability of going to the hospital when sick, *β*_0_ is the intercept item, *Insurance*_*i*_ indicates the status of whether the individual participates in medical insurance, and *β*_1_ indicates the coefficient of the impact of the medical insurance participation status on whether an individual would go to the hospital. *Person*_*i*_ indicates a series of personal characteristic variables, including gender, age, ethnicity, hukou type, marital status, and education level, *β*_2_ indicates the coefficient of the impact of individual characteristics on whether the individual would go to the hospital. *Control*_*i*_ indicates other control variables, including range of migration, household income, personal economic source, number of friends, daily exercise time, physical examination status, inflowing provinces, etc. *β*_3_ indicates the influence of other control variables on whether an individual would go to the hospital, and *ε*_*i*_ is a random disturbance term.

Meanwhile, PSM was used to examine the effects of institutional differences and geographical disparity on the utilization of health services by the floating elderly population. We assume that floating elderly population *i* is the treatment group that participates in the BMISUE, and floating elderly population *j* is the control group that participates in the BMISURR. An individual *j* in the control group needs to be found so that the observable variables of *i* and *j* can be nearly matched, that is *x*_*i*_ = *x*_*j*_; however, exact matching is not easy to achieve, so a single index, that is, the “tendency score” is used for matching.

The propensity score of *i* is the conditional probability of *i* entering the processing group under *x*_*i*_ and is given as *P*(*x*_*i*_) = *P*(*D*_*i*_ = 1|*x* = *x*_*i*_). For each individual in the control group, a propensity score is used as a distance function for matching, and the average utility of each individual is obtained as matching estimators.

## Results

### Regression results of the effect of the institutional difference in the medical insurance system on the equity of the utilization of health services by the floating elderly population

As mentioned in the introduction, under the current medical insurance system in China, different medical insurance systems may have an impact on the utilization of health services by the floating elderly population population. Table 4 reports the logit regression results for the effects of the different medical insurance systems on the equity of health services for the floating elderly population in China.

**Table 4:** Logit regression results of the impact of different medical insurance system on the utilization of health services by the floating elderly population

Variables		(1)		(2)		(3)		(4)	
			the Average Marginal Effect		the Average Marginal Effect		the Average Marginal Effect		the Average Marginal Effect
	Medical insurance	− 0.157*	− 0.0383*	− 0.147*	− 0.0355*	− 0.0912	− 0.0215		
		(0.0810)	(0.0197)	(0.0823)	(0.0199)	(0.0849)	(0.0200)		
	No medical insurance							0.0629	0.0148
								(0.0883)	(0.0208)
	BMISUE							−0.124	−0.0292
								(0.102)	(0.0239)
	Gender			−0.124*	−0.0299*	− 0.105	− 0.0248	− 0.103	− 0.0243
				(0.0644)	(0.0156)	(0.0677)	0.0160	(0.0678)	(0.0160)
	Age			0.00957*	0.00232*	0.00718	0.00169	0.00737	0.00174
				(0.00553)	(0.00134)	(0.00602)	0.00142	(0.00603)	(0.00142)
	Ethnicity			−0.142	−0.0343	−0.0845	−0.0199	− 0.0838	− 0.0198
				(0.106)	(0.0256)	(0.109)	(0.0256)	(0.109)	(0.0257)
Household registration type (Hukou)	Rural hukou			−0.0949	−0.0230	0.0479	0.0113	0.0128	0.00301
				(0.0733)	(0.0177)	(0.0873)	(0.0206)	(0.0919)	(0.0217)
	Rural to resident hukou			−0.0500	−0.0121	−0.0285	−0.00671	− 0.0705	−0.0166
				(0.279)	(0.0676)	(0.284)	0.0670	(0.288)	(0.0680)
	Nonrural to resident hukou			0.487	0.118	0.306	0.0721	0.334	0.0787
				(0.382)	(0.0924)	(0.393)	(0.0926)	(0.394)	(0.0928)
Marital Status	Single			−0.359	− 0.0869	− 0.210	− 0.0495	−0.217	− 0.0511
				(0.331)	(0.0799)	(0.337)	(0.0794)	(0.337)	(0.0794)
	Married			−0.202**	−0.0490**	−0.187**	− 0.0440**	−0.184**	− 0.0434**
				(0.0812)	(0.0196)	(0.0825)	(0.0194)	(0.0826)	(0.0194)
	Education Level			0.0674**	0.0163**	0.0679**	0.0160**	0.0730**	0.0172**
				(0.0322)	(0.00779)	(0.0337)	(0.00792)	(0.0339)	(0.00798)
Range of Migration	Interprovince					0.317***	0.0748***	0.315***	0.0742***
						(0.0811)	(0.0190)	(0.0810)	(0.0190)
	Intercity					0.0353	0.00833	0.0366	0.00862
						(0.0827)	(0.0195)	(0.0827)	(0.0195)
	Monthly household income					2.15e-05***	5.08e-06***	2.15e-05***	5.06e-06***
						(7.06e-06)	1.66e-06	(7.00e-06)	(1.64e-06)
Main Source of Income	Income from employment					−0.258***	−0.0609***	−0.258***	−0.0609***
						(0.0959)	(0.0226)	(0.0959)	(0.0225)
	Pension/savings					0.0538	0.0127	0.0901	0.0212
						(0.0984)	(0.0232)	(0.102)	(0.0241)
	Others					−0.0518	− 0.0122	− 0.0537	− 0.0127
						(0.162)	(0.0381)	(0.162)	(0.0381)
	No. of friends of residence					0.00381	0.000899	0.00390	0.000920
						(0.00285)	(0.000672)	(0.00285)	(0.000672)
	Daily exercise time					−0.000841	−0.000198	−0.000800	−0.000188
						(0.000723)	(0.000170)	(0.000723)	(0.000170)
	Physical examination					0.223***	0.0527***	0.225***	0.0530***
						(0.0657)	(0.0154)	(0.0657)	(0.0154)
	Self-rated health					0.0624	0.0147	0.0626	0.0148
						(0.0496)	(0.0117)	(0.0496)	(0.0117)
	Province of residence					0.0130***	0.00307***	0.0130***	0.00307***
						(0.00185)	(0.000426)	(0.00185)	(0.000426)
	Constant	−0.185**	−0.185**	−0.580	−0.580	−1.567***	−1.567***	−1.651***	−1.651***
		(0.0739)	(0.0739)	(0.431)	(0.431)	(0.521)	(0.521)	(0.519)	(0.519)
	Observations	4484		4484	4484	4481		4481	
	Pseudo-R^2^	0.0006		0.0058		0.0247		0.0249	

1) Columns (1)–(3) divides medical insurance into two categories: “with medical insurance” and “no medical insurance,” and “no medical insurance” is the baseline variable. Column (4) divides medical insurance into three categories: “BMISUE”, “BMISURR”, and “no medical insurance”, and “BMISURR” is the baseline variable. 2) The baseline variables for household registration type, marital status, distance from place of origin, and main source of income are “nonrural hukou,” “divorced or widowed,” “intercity,” and “other members of the family”, respectively. 3) The robust standard errors are reported in parentheses. 4) *** *p* < 0.01, ** *p* < 0.05, * *p* < 0.1

Columns (1)–(3) in Table 4 classify the medical insurance status of the floating elderly population into those with medical insurance and those without medical insurance. Column (1) reports the regression results when there is no control variable. Column (2) reports the regression results when controlling the individual characteristics of gender, age, hukou, ethnicity, education level, etc. Column (3) reported the regression results when controlling the individual characteristics and the self-rated health, income source, physical examination, daily exercise and other conditions. The results show that when only the individual characteristics are controlled, the floating elderly population with medical insurance has a 3.83% lower probability of going to the hospital than the population without medical insurance,. However, when variables such as self-rated health and main source of income are controlled, the floating elderly population with medical insurance has a 2.15% lower probability of going to the hospital than those without medical insurance,. However, this difference is not statistically significant (*P* = 0.283). That is, participate in medical insurance does not affect the behavior of the floating elderly population.

Column (4) in Table 4 divides the medical insurance status of the floating elderly population into three categories: BMISUE, BMISURR, and non-medical insurance. The baseline variable is “BMISURR” for identifying the impact of different types of medical insurance on the behavior of individuals when they are sick. The regression results show that the average marginal effect coefficient of the variable “BMISUE” is − 0.0292, which is not statistically significant (*P* = 0.222), indicating that compared with the floating elderly population participating in the BMISURR, the probability that the floating elderly population participating in the BMISUE will go to the hospital is not significantly improved.

The regression results of other variables showed that the more educated an individual is, the more likely he or she will to go to the hospital. The range of migration, monthly household income and physical examination all significantly affect the probability of using health services. However, the household registration type is not statistically significant for the use of health services.

### Regression results of the effect of the geographical disparity in medical insurance on the equity of the utilization of health services by the floating elderly population

As mentioned in the introduction, under the current medical insurance system in China, the geographical disparity within the same system and between the various systems may also affect the utilization of health services by the elderly population. Table 5 reports the logit regression results of the impact of differences in the medical insurance system on the utilization of health services by the floating elderly population in China.

**Table 5:** Logit regression results of the geographical disparity of the medical insurance system for the utilization of health services by the floating elderly population

Variables		(1)		(2)		(3)	
			the Average Marginal Effect		the Average Marginal Effect		the Average Marginal Effect
Medical insurance	(in residence places)	0.316***	0.0743***				
		(0.104)	(0.0243)				
BMISURR	(in residence places)			0.208*	0.0480*		
				(0.119)	(0.0275)		
BMISUE	(in residence places)					0.438*	0.103*
						(0.234)	(0.0544)
	No medical insurance	0.133	0.0313				
		(0.0860)	(0.0202)				
	Gender	−0.101	−0.0237	−0.177*	−0.0410*	− 0.0140	−0.00328
		(0.0678)	(0.0159)	(0.0915)	(0.0211)	(0.136)	(0.0318)
	Age	0.00741	0.00174	0.0101	0.00234	0.0153	0.00358
		(0.00602)	(0.00141)	(0.00848)	(0.00196)	(0.0110)	(0.00257)
	Ethnicity	−0.0645	− 0.0152	− 0.307**	− 0.0709**	− 0.277	− 0.0650
		(0.109)	(0.0256)	(0.143)	(0.0329)	(0.236)	(0.0551)
Household registration type (Hukou)	Rural hukou	0.0530	0.0125	−0.0667	− 0.0154	− 0.0203	− 0.00476
		(0.0872)	(0.0205)	(0.120)	(0.0277)	(0.224)	(0.0526)
	Rural to resident hukou	−0.0204	− 0.00479	− 0.441	− 0.102	1.453*	0.340*
		(0.286)	(0.0674)	(0.378)	(0.0873)	(0.821)	(0.191)
	Nonrural to resident hukou	0.310	0.0730			0.417	0.0977
		(0.387)	(0.0910)			(0.420)	(0.0981)
Marital Status	Single	−0.226	−0.0531	0.0857	0.0198		
		(0.340)	(0.0799)	(0.412)	(0.0952)		
	Married	−0.185**	−0.0434**	−0.105	− 0.0243	−0.411**	− 0.0964**
		(0.0826)	(0.0194)	(0.109)	(0.0251)	(0.190)	(0.0441)
	Education level	0.0692**	0.0163**	0.0696	0.0161	0.0539	0.0126
		(0.0337)	(0.00791)	(0.0510)	(0.0118)	(0.0567)	(0.0132)
Range of Migration	Interprovince	0.314***	0.0738***	0.264**	0.0610**	0.308*	0.0721*
		(0.0813)	(0.0190)	(0.104)	(0.0239)	(0.165)	(0.0383)
	Intercity	0.0345	0.00813	−0.0257	− 0.00593	− 0.0677	− 0.0159
		(0.0828)	(0.0195)	(0.106)	(0.0245)	(0.163)	(0.0383)
	Monthly household income	2.26e-05***	5.31e-06***	3.68e-05***	8.50e-06***	5.46e-06	1.28e-06
		(7.27e-06)	(1.70e-06)	(8.36e-06)	(1.91e-06)	(1.06e-05)	(2.49e-06)
Main Source of Income	Income from employment	−0.257***	− 0.0605***	− 0.179	− 0.0414	− 0.340	−0.0796
		(0.0960)	(0.0225)	(0.113)	(0.0262)	(0.743)	(0.174)
	Pension/savings	0.0687	0.0162	0.0499	0.0115	0.196	0.0459
		(0.0986)	(0.0232)	(0.124)	(0.0287)	(0.657)	(0.154)
	Other	−0.0642	−0.0151	0.0361	0.00833		
		(0.162)	(0.0382)	(0.190)	(0.0440)		
	No. of friends of residence	0.00366	0.000862	0.00386	0.000891	0.00289	0.000678
		(0.00286)	(0.000674)	(0.00409)	(0.000945)	(0.00457)	(0.00107)
	Daily exercise time	−0.000886	−0.000209	0.000795	0.000184	−0.00404***	− 0.000946***
		(0.000724)	(0.000170)	(0.000956)	(0.000221)	(0.00143)	(0.000330)
	Physical examination	0.207***	0.0487***	0.252***	0.0583***	−0.0940	−0.0220
		(0.0661)	(0.0155)	(0.0877)	(0.0201)	(0.131)	(0.0307)
	Self-rated health	0.0666	0.0157	0.00942	0.00218	0.0374	0.00875
		(0.0497)	(0.0117)	(0.0641)	(0.0148)	(0.104)	(0.0243)
	Province of residence	0.0126***	0.00297***	0.0122***	0.00283***	0.0162***	0.00380***
		(0.00185)	(0.000428)	(0.00245)	(0.000555)	(0.00371)	(0.000838)
	Constant	−1.742***	−1.742***	−1.562**	−1.562**	−1.554	−1.554
		(0.519)	(0.519)	(0.714)	(0.714)	(1.175)	(1.175)
	Observations	4481		2631		1109	
	Pseudo-R^2^	0.0262		0.0319		0.0352	

1). Column (1) divides medical insurance into three categories: “medical insurance (in residence places),” “medical insurance (in non-residence places)” and “No medical insurance,” and “medical insurance (in non-residence places)” is the baseline variable. Clumn (2) divides BMISURR into two categories: “BMISURR (in residence places)” and “BMISURR (in non-residence places)”, and “BMISURR (in non-residence places)” is the baseline variable. Column (3) divides BMISUE into two categories: “BMISUE (in residence places)” and “BMISUE (in non-residence places)”, and “BMISUE (in non-residence places)” is the baseline variable. 2), The baseline variable for household registration type, marital status, flow range, the main economic are “Nonrural hukou,” “divorce or widowed,” “intercity”, “other members of the family”, respectively. 3),The robust standard errors are reported in parentheses. 4), *** *p* < 0.01, ** *p* < 0.05, * *p* < 0.1

Column (1) in Table 5 divides the medical insurance participation status into three types: “medical insurance (in residence places)”, “medical insurance (in non-residence places)” and “no medical insurance”. The baseline variable is “medical insurance (in non-residence places)” for identifying the impact of the geographical disparity in medical insurance on the probability that an individual in the floating elderly population visits the hospitals when sick. The results show that the average marginal effect coefficient of the variable “medical insurance (in residence places)” is − 0.0743, which is significant at the level of 1%. This indicates that compared with the floating elderly population participating in “medical insurance (in non-residence places)”, the probability that an individual in the floating population who participates in medical insurance (in residence places) goes to the hospital increases by 7.43%. In other words, participating in medical insurance in the place of residence improves the utilization of health services by the floating elderly population.

We further analyzed the impact of the geographical disparity of the same medical insurance system on the utilization of health services by the floating elderly populations. Column (2) in Table 5 reports the impact of geographical disparity in the participation of BMISURR on the behavior of the floating elderly population. The results show that the average marginal effect coefficient of the variable “ BMISURR (in residence places)” is 0.0480, which is statistically significant, indicating that relative to the floating elderly population participating in “BMISURR (in non-residence places)”, the probability that an individual participating in “BMISURR (in residence places)” will go to the hospital increases by 4.80%.

Column (3) reports the impact of the geographical disparity in the participation of the BMISUE on the behavior of the floating elderly population. The results show that the average marginal effect coefficient of the variable “BMISUE (in residence places)” is 0.103, which is statistically significant, indicating that relative to the floating elderly population participating in “BMISUE (in non-residence places)”, the probability that an individual participating in “BMISUE (in residence places)” will go to the hospital increases by 10.30%.

From the regression results in Table 5, in China, when the floating elderly participate in medical insurance outside the place of residence, they are less likely to see a doctor when they are sick. Even for those with the same kind of medical insurance, regardless of whether it is the BMISUE or the BMISURR, the participation of the floating elderly population in their place of residence will significantly increase the probability of seeing a doctor, while participation outside the place of residence will significantly reduce the probability of seeing a doctor. That is, even if individuals in the floating elderly population participate in a type of medical insurance, the geographical difference between the place of participation and the place of residence will lead to the inequitable use of health services.

### Further testing of the results using the PSM method

To further test the robustness of the results, we use the PSM method to analyze the impact of institutional differences and geographical disparity on the utilization of health services by the floating elderly population. Table 6 reports the average treatment effect on the treated (ATT) according to the of PSM results.

**Table 6:** PSM results for the impact of institutional differences and geographical segmentation on the use of health services by the floating elderly population

	Treated	Controls	Difference	S.E.	T-stat
(1) ATT	0.436	0.470	−0.0344	0.0301	−1.14
(2) ATT	0.487	0.410	0.0770	0.0281	2.74
(3) ATT	0.527	0.410	0.117	0.0613	1.91
(4) ATT	0.471	0.406	0.0643	0.0319	2.02

1) The sample only includes the elderly population that participated in medical insurance. The elderly population that did not participate in medical insurance was excluded. 2) Column (1) reports the impact of different medical insurance systems on the utilization of health services by the floating elderly; the treatment group is “BMISUE”, and the control group is “BMISURR.”3) Column (3) and Column (4) report the impact of participating in the BMISUE/BMISURR on the utilization of health services by the floating elderly population in different regions. 4) In the PSM process, a pair of 4 matches in the caliper is used, and the caliper is set to 0.01

Column (1) of Table 6 reports the impact of different medical insurance systems on the utilization of health services by the floating elderly; the treatment group is “BMISUE”, and the control group is “BMISURR.” The results show that the coefficient of the ATT is − 0.0344, and the T-statistic is − 1.14, which is not statistically significant. This shows that participating in different medical insurance systems has no significant influence on the utilization of health services by the floating elderly population.

Column (2) reports the impact of participating in medical insurance on the use of health services in different areas (residence and non-residence), the treatment group is “medical insurance (in residence places)” and the control group is “medical insurance (in non-residence places)”. The results show that the coefficient of the ATT is 0.077 and the value of the T-statistic was 2.74. This shows that participating in medical insurance in the place of residence increases the probability of seeing a doctor by 7.7% compared with participating in medical insurance at the place of non-residence.

Column (3) reports the impact of participating in the BMISUE on the utilization of health services by the floating elderly population in different regions (residence and non-residence), including the treatment group “BMISUE (in residence places)” and control group “BMISUE (in non-residence places)”. The result show that the ATT coefficient is 0.117 and the value of T-statistic is 1.91. This shows that participating in the BMISUE (in residence places) increases the probability of seeing a doctor by 11.7% compared with participating in the BMISUE (in non-residence places).

Column (4) reports the impact of participating in the BMISURR in different regions (residence and non-residence) on the utilization of health services by the floating elderly population.. The treatment group is “BMISURR (in residence places)” and the control group is “BMISURR (in non-residence places).” The ATT coefficient is 0.0643, and the value of T-statistic is 2.02. This shows that participating in the BMISURR (in residence places) increases the probability of seeing a doctor by 6.43% compared with participating in the BMISURR (in non-residence places)..

The results of PSM in Table 6 are consistent with the results in Tables 4 and 5. First, participation in the BMISUE or in the BMISURR does not affect the utilization of health services by the floating elderly population. The institutional differences have not caused inequity in the use of health services by China’s floating elderly population. Second, for those participating in the same medical insurance system, participation in different regions will significantly affect the utilization of health service resources. Regardless of whether the BMISUE or BMISURR is considered, being insured in the place of residence will substantially improve the utilization of health services by the floating elderly population. This geographical disparity leads to inequitable use of health services by the floating elderly population.

## Discussion

### The impact of the institutional difference in the medical insurance system on the utilization of health services by the floating elderly

The results in Tables 4 and 6 show that compared with the BMISURR, participating in the high level of protection of the BMISUE did not significantly improve the utilization of health services by the floating elderly population.

Table 7 compares the BMISUE and BMISURR regarding financing channels, fundraising levels and reimbursement rates. Table 7 shows that the two types of medical insurance are different regarding population, financing mode and reimbursement status. On the whole, the BMISUE is a high-paying, high-reimbursement medical insurance system and the BMISURR is a low-paid, low-reimbursement medical insurance system. From the perspective of the per capita funding level, the fundraising level of the BMISURR is not only far lower than that of the BMISUE (the fundraising level of the BMISUE was 7.28 times that of the BMISURR in 2014), but it is highly subsidized by the government, and the self-paid portion only accounts for approximately 13%. From the perspective of the hospitalization reimbursement and reimbursement capping of the two systems, the ratio of hospitalization reimbursement for the two systems is relatively close, on average, which means that the level of protection of the BMISUE is not proportionally higher than that of the BMISURR. Therefore, this institutional difference does not affect the utilization of health services by the floating elderly population.

**Table 7:** Comparison of basic medical insurance between BMISUE and BMISURR

	BMISUE	BMISURR	
		BMISUR	NRCMS
Establishment time	1998	2007	2003
Covering	all urban employers and their employees; informal employment and flexible employment	all nonemployed urban residents: students in primary and secondary schools, children and other nonemployed urban residents	all rural residents
Coordination level	city-level or county-level coordination	city-level coordination	county-level coordination
Fund-raising channels	the employer and the employees jointly pay, in which the employer’s contribution rate is 6% of the employee’s salary	the individual contribution rate is 2% of the employee’s salary	The individual payment and the government subsidy are combined
Per capita funding level (2014)	3820.1 yuan	524.4 yuan (government subsidy: 461.2 yuan, personal burden: 63.2 yuan)	410.9 yuan (government subsidy: 355.2 yuan, personal burden: 55.7)
No. of participants (2014)	282.96 million	314.59 million	737.00 million
Average hospitalization reimbursement rate in the catalogue (2014)	80%	75%	70%
Reimbursement cap	6 times the average social wage of employees in the city	6 times the disposable income of local people	6 times the per capita net income of local farmers

The per capita funding level, the number of participants and the per capita funding expenditure data are from the National Bureau of Statistics. The rest of this form is compiled from national policy documents

### The impact of geographical disparity in the medical insurance system on the utilization of health services by the floating elderly population

The results of Table 5 and Table 6 show that the geographical segmentation of the medical insurance system significantly affects the equity of health service utilization of the floating elderly population, whether the BMISUE or the BMISURR is considered. The health service utilization rate of those participating in the medical insurance system in the place of residence is higher than the utilization rate of those participating in a different place. The geographical segmentation of medical insurance creates inequitable use of health services for the floating elderly. To explain this inequity, we found that the lower level of overall planning by the current medical insurance system is the key reason for this city-county level coordination.

Under the current system design, where the prefecture-level city or county-level city is the basic pooling unit to prevent the unreasonable waste of medical resources, reduce the occurrence of moral hazard and reduce the system expenditure, the BMISUE and BMISURR encourage the insured to use the health resources in the place of insurance when receiving medical services [20]. In the same medical insurance system, if an individual is not insured in the same place where the medical service is used, that is, if the insured receives medical services in a place other than the place of participation, the reimbursement is lower than it would be if medical services ad been received in the place of participation. For example, the administrative department of Henan Province of China stipulates the proportion of reimbursement for the BMISURR: for those enrolled in a municipal-level area of the province who receive medical services in a participating city in Henan Province, the reimbursement standard is 500 yuan, and the reimbursement rates are 55% (for expenses of 500–3000 yuan) and 75% (for expenses over 7000 yuan), while for those who receive medical services in Henan Province but not in a participating city, the reimbursement standard increases to 600 yuan, and the reimbursement rates are reduced to 53% (for 600–4000 yuan) and 72% (for over 7000 yuan), and for those receiving medical services outside Henan Province, the deductible for reimbursement is 1500 yuan, and the reimbursement rates are reduced to 50% (for expenses of 1500–7000 yuan) and 68% (for expenses over 7000 yuan).

Second, when medical service expenses are incurred in different places, since no medical information sharing system has been established, the medical information data cannot be shared. The settlement and reimbursement of expenses incurred in different places require the insured to provide various certification materials, and the reimbursement procedure is very cumbersome, and in some cases, reimbursement is impossible. The relatively low level of compensation for health services and the inconvenience of reimbursement procedures in different places have reduced the utilization of health services by the floating elderly, resulting in inequitable use of the resources of the medical insurance system.

There are some research limitations in this paper. For example, first, subject to data restrictions, we use the subjective responses of the insured concerning their personal health service visit rate as an indicator to assess the health service utilization rate of the insured. Therefore, we do not rule out the interference of the subjective factors of the respondent; however, a satisfactory solution cannot be found in the relevant official data currently published in China. In the future, we will seek to adopt a more objective evaluation method to further test the research conclusions. Second, only the issue of equity in the utilization of health services by the floating elderly population is discussed due to limited data availability. In the future, we will collect more data to address the issue of the equity of medical services for the entire floating population.

## Conclusions

From the analytical results, it can be concluded that under the current policy, although there is a significant difference in the payment level and reimbursement level of the BMISUE and the BMISURR, the differences in the system do not have a significant impact on the equity in the health services utilization by the floating elderly population.

Moreover, the geographical disparity of the system resulting from the lower-level design of the current system significantly affects the equity of the utilization of health services by the elderly. Therefore, this paper posits that although the Chinese government has established a medical insurance system covering all citizens, which has improved the overall health of all citizens to a certain extent, the current medical insurance system still has certain defects in design. In the future, the Chinese government might improve the medical insurance system design, and the most important thing is to improve the overall level of the medical insurance system and place the pooling districts at the lower administrative level to completely eliminate the inequity brought by problems at the city and county levels.

According to the results of this paper, it finds that the equity in the health service utilization by the floating elderly population can be improved from the following two aspects. On the one hand, we can accelerate the construction of basic medical insurance information systems, encourage interregional information sharing, resource sharing and network settlement, and encourage all localities to actively explore the use of social service resources to participate in and establish cross-regional settlement platforms and enhance the convenience to residents. On the other hand, it may be necessary to improve the pooling level of the medical insurance system and achieve central planning to eliminate the inequitable utilization of health services caused by geographical disparity.

## Data Availability

The datasets generated and/or analyzed during the current study are available in the 2015 China Migrants Dynamic Survey (CMDS) repository (from http://www.chinaldrk.org.cn/wjw/#/home/) .

## Abbreviations

ATT: Average treatment effect on the treatment
BMISUE: Basic Medical Insurance System for Urban Employees
BMISUR: Basic Medical Insurance System for Urban Residents
BMISURR: Basic Medical Insurance System for Urban and Rural Residents
CHNS: China Health Nutrition Survey
CMDS: China Migrants Dynamic Survey
NRCMS: New Rural Cooperative Medical System
PSM: Propensity score matching
